# Odontogenic ameloblast-associated protein (ODAM) in gingival crevicular fluid for site-specific diagnostic value of periodontitis: a pilot study

**DOI:** 10.1186/s12903-018-0609-0

**Published:** 2018-08-24

**Authors:** Hye-Kyung Lee, Soo Jin Kim, Young Ho Kim, Youngkyung Ko, Suk Ji, Joo-Cheol Park

**Affiliations:** 1 Departments of Oral Histology-Developmental Biology & Dental Research Institute, School of Dentistry, Seoul National University, 101 Daehagro, Chongro-gu, Seoul, 110-749 South Korea; 2 Office of Biostatistics, Institute of Medical Sciences, Ajou University School of Medicine, Suwon, South Korea; 3 Department of Orthodontics, Institute of Oral Health Science, Ajou University School of Medicine, Suwon, South Korea; 4 Department of Periodontics, College of Medicine, Seoul St Mary’s Hospital, The Catholic University of Korea, Seoul, South Korea; 5 Department of Periodontics, Institute of Oral Health Science, Ajou University School of Medicine, 164, World cup-ro, Yeongtong-gu, Suwon, South Korea

**Keywords:** Odontogenic ameloblast-associated protein (ODAM), Periodontitis, Gingival crevicular fluid, Biomarker

## Abstract

**Background:**

Odontogenic Ameloblast-Associated Protein (ODAM) in gingival crevicular fluid (GCF) can provide evidence of the detachment of junctional epithelium from the tooth surface by periodontitis. This study sought to investigate the ability of ODAM to reflect the severity of periodontitis at a site-specific level; thus whether there was a relationship between clinical diagnostic parameters and the value of ODAM in GCF was analyzed.

**Methods:**

Eight periodontitis patients with various severities were enrolled, and the clinical parameters and samples of GCF were obtained from 44 to 60 sites of each subject. The ODAM concentration was quantified by enzyme-linked immunosorbent assay. Correlation analyses between clinical parameters and ODAM values and unadjusted and adjusted (linear) mixed model analyses were performed. The accuracy of ODAM to reflect sites having a probing depth (PD) ≥ 5 mm and a positive bleeding on probing (BOP) was evaluated by receiver-operating characteristic analysis.

**Results:**

A total of 424 GCF samples were collected. The mean ODAM concentration from each patient varied from 0.2 to 1.52 ng/ml. Correlations between PD or clinical attachment level (CAL) and ODAM values were found (*p* <  0.0001). An adjusted linear mixed model showed that PD or CAL were associated with ODAM values (*p* <  0.05). The area under the curve of ODAM, which reflected sites with PD ≥ 5 mm and positive BOP, was 0.661 (*p* <  0.0001).

**Conclusion:**

This result shows the possibility of GCF ODAM as a site-specific biomarker for periodontal tissue destruction.

## Background

Periodontitis is chronic inflammation of the periodontium caused by the host’s immune response to subgingival bacterial biofilm, which can lead to the irreversible destruction of connective tissue and bone. The gingival epithelium provides the first line of defense against invading bacteria, forming barriers between plaque bacteria and gingival tissue [1].

The integrity of the junctional epithelium (JE) is therefore essential for maintaining a healthy periodontium. Immunologically, the JE plays a role in protection as a physical, chemical, and immunological barrier to protect the underlying gingival connective tissue and bone from exposure to bacteria and their products [1]. It has a specialized epithelial structure that attaches the gingival soft tissue to the tooth surface consisting of an internal basal lamina and hemidesmosomes [2]. During the progress into periodontitis, JE detaches from the tooth surface and migrates apically and laterally toward the space being formed through connective tissue destruction [3, 4]. Therefore, the detachment of JE from the tooth surface is regarded as the hallmark in the progression of periodontitis.

In previous study, we reported that odontogenic ameloblast-associated protein (ODAM) was extruded from the JE following the onset of JE attachment loss and was detected in gingival crevicular fluid (GCF), and proposed that ODAM could be used as a biomarker of periodontitis and peri-implantitis [5]. ODAM is a secretory calcium-binding phosphoprotein expressed by ameloblasts during the maturation stage of enamel formation, and its expression persists in the reduced enamel organ and JE of gingiva at the erupted tooth [6]. It was known that ODAM is implicated in the adhesion of epithelial cells to tooth surfaces [5, 7]. Wazen et al. recently showed that ODAM plays a role in maintaining the integrity of the JE and gingival healing using an ODAM knockout mouse model [7]. We also identified that the adhesion of the JE to the tooth surface is regulated via fibronectin/laminin-integrin-ODAM-ARHGEF5-RhoA signaling, and ODAM-mediated RhoA signaling resulted in actin filament rearrangement [5].

The diagnosis of periodontitis is currently performed using radiography and clinical measurements, such as probing depth (PD), clinical attachment level (CAL), bleeding on probing (BOP), suppuration and mobility [8]. These traditional clinical measurements not only reflect a history of periodontal disease but also help to determine prognosis, however there is an unmet need for an easily accessible test showing disease activity to diagnose periodontitis.

Extensive research has been carried out on GCF and saliva components that might serve as potential diagnostic markers for periodontitis [9, 10]. The collection of saliva is relatively simple, safe, and non-invasive, so saliva can be used as a point-of-care diagnostic tool for periodontitis [10]. However, saliva is limited in detecting disease activity at each individual tooth site, and so traditional clinical measurements should be taken to detect the tooth site affected by periodontitis, even if one subject is diagnosed with periodontitis using saliva. In this respect, the diagnosis using GCF can be useful to diagnose the disease at specific sites. GCF contains a large number of proteins and peptides liberated from the underlying tissues [9, 11, 12], so the analysis of the GCF components can reflect the disease status of individual tooth sites.

This pilot study sought to investigate the ability of ODAM to reflect the severity of periodontitis at a site-specific level. The analysis of ODAM values from single-site GCF may enable the clinician to distinguish between healthy sites and those affected by periodontitis. For this purpose, we performed a cross-section study of ODAM values in GCF as well as of corresponding clinical parameters in periodontitis patients and analyzed whether there was a relationship between clinical diagnostic parameters and the concentration of ODAM in GCF.

## Methods

### Patient population

Eight periodontitis patients having both sites with PD ≤ 3 mm and diseased sites with PD ≥ 4 mm were participated in this study. The study protocol was approved by the Korea University Anam Hospital, Seoul, Korea (IRB no. ED13162) and participants provided written informed consent to participate in this study. Patients with at least 25 teeth had to have at least 5 teeth having site with PD ≥ 4 mm, and had not received periodontal treatment for the last 2 years. According to the exclusion criteria, all participants had no history of systemic disease, which could influence the prognosis of periodontitis, no smoking, no untreated caries, no orthodontic appliances, were not pregnant/breast-feeding, and were not treated with medications (antibiotic, antimicrobial, and/or anti-inflammatory drugs) during the 6 months before examination and sampling.

### Clinical examination and gingival crevicular fluid (GCF) collection

Panoramic X-rays and plaque index (PI) scores were recorded for all patients. Prior to measuring PD, GCF was sampled because the gingival bleeding during the measurement of PD can be sucked in GCF sampling strips. GCF samples were obtained from teeth of one quadrant that contained the teeth showing the most severe marginal bone loss on the panoramic X-ray view and the contralateral quadrant of the opposite jaw. GCF samples were obtained from four sites of each tooth (mesiobuccal, mesiolingual, distobuccal, and distolingual sites) using absorbent paper strips (ORAFLOW, Smithtown, NY, USA). Supragingival plaque on the tooth surface was carefully removed with curettes, avoiding bleeding from the gingiva, and each tooth site was gently dried for 10 s with compressed air. Paper strip was inserted carefully into the gingival sulcus/pocket until mild resistance was felt and left in place for 30 s. There was no strip that was saturated by GCF within the 30 s collection timeframe. In rare cases, the strip was saturated by saliva that was not sufficiently blocked, and those strips were discarded. Then, the strip was transferred into a microtube containing 100 μl of phosphate-buffered saline, and the microtubes were stored at − 80 °C until analyzed. Red-stained strips that were visibly contaminated with blood were discarded. After obtaining samples of GCF, clinical parameters of PD, CAL, and modified sulcus bleeding index (mSBI) [13] were recorded from the same four sites of each tooth. Therefore, a total of 428 sites were analyzed by collecting 44 to 60 recordings of clinical parameters and samples of GCF from every 11–15 teeth of each patients. For sites where GCF was not sampled, clinical parameters were also recorded at four sites per tooth, including BOP instead of mSBI.

### Enzyme-linked immunosorbent assay (ELISA)

The microtubes containing the paper strip were thawed and shaken on an ELISA plate shaker for 60 min and then centrifuged at 13,200 rpm for 3 min at 4 °C. The supernatants were used for ELISA analysis. The total levels of ODAM in GCF samples were assayed using an ODAM ELISA kit according to the instructions of the manufacturer (Cusabio Biotech, Wuhan, China). The ODAM levels were calculated from standard curves and expressed as the concentration calculated from the ELISA assay itself (ng/ml per 30 s sample).The minimum detection limit was 0.002 ng/ml for ODAM.

### Statistical analysis

Mean values for PD, CAL, PI and mSBI and percentages of BOP were calculated for each patient. Percentages of sites showing PD ≤ 3 mm, PD, 4–5 mm, and PD ≥ 6 mm were also calculated for each patient. Spearman’s rank correlation analyses between PD, CAL, or mSBI and ODAM values were performed. The ODAM values were divided into four groups according to the degree of clinical parameters and the differences in ODAM values among groups were compared using ANOVA. Unadjusted and adjusted (linear) mixed model analysis was performed using the non-parametric test. Adjusted mixed models, adjusting the random effects of subject, and the fixed effects of age, sex, mSBI, and PI were considered to identify the linear association between PD, CAL, or mSBI and ODAM values. The accuracy of ODAM to reflect sites having a probing depth (PD) ≥ 5 mm and a positive BOP was evaluated by receiver-operating characteristic (ROC) analysis and areas under the receiver operating characteristic curve (AUC) were calculated to compare the predictive ability of the indices, and optimal cut-off values were determined using the Youden index to maximize the sum of sensitivity and specificity. On the basis of the AUC statistic, the diagnostic test can be either non-informative (AUC = 0.5), less accurate (0.5 < AUC ≤ 0.7), moderately accurate (0.7 < AUC ≤ 0.9), highly accurate (0.9 < AUC < 1), or perfect (AUC = 1) [14]. All statistical analyses were performed using SPSS version 23.0 (SPSS, Inc., Chicago, IL, USA) and MedCalc version 16.8 (MedCalc Mariakerke, Belgium). The results were considered statistically significant when *p*-values were less than 0.05.

## Results

### Characteristics of full mouth and sampled sites

Eight periodontitis patients (4 males and 4 females) with a mean age of 57 (range, 44–74) years were enrolled in this study. Table 1 presents the periodontal status of the full mouth and the selected sites for GCF sampling. Each patient had at least 18% (and up to 68.5%) of sites with a PD ≥ 4 mm, and 60% (and up to 88.7%) of sites were BOP-positive. The percentages for sites with PD ≤ 3 mm among the sampling sites were calculated between 32.7 and 83.7% and that for sites with PD ≥ 6 mm were calculated between1.9 and 25.4%. The mean PD of the sampling sites ranged from 3.14 ± 0.13 mm (Mean ± SD) to 4.42 ± 0.27 mm in individual patients (Table 1).

**Table 1:** Clinical characteristics of full mouth and GCF sampling sites

Patient code	Number of teeth	Number of sites	PD (Mean ± SD)	% of sites with PD ≤ 3 mm	% of sites with PD 4–5 mm	% of sites with PD ≥ 6 mm	CAL (Mean ± SD)	PI (Mean ± SD)	BOP %	mSBI (Mean ± SD)
Full mouth clinical data										
S1	25	100	3.12 ± 0.07	82.1	14.9	3.0	3.60 ± 0.1	0.70 ± 0.50	60.0	
S2	28	112	4.05 ± 0.13	31.5	66.2	2.3	4.77 ± 0.18	0.85 ± 0.07	87.5	
S3	28	112	4.25 ± 0.26	44.2	30.2	25.6	5.11 ± 0.3	0.40 ± 0.09	61.0	
S4	28	112	4.17 ± 0.24	48.2	42.3	9.5	4.40 ± 0.25	0.56 ± 0.07	66.7	
S5	28	112	4.56 ± 0.24	42.3	41.5	16.2	4.56 ± 0.24	0.04 ± 0.03	81.3	
S6	27	108	4.21 ± 0.22	41.3	48.5	10.2	4.75 ± 0.31	0.56 ± 0.07	78.6	
S7	25	100	3.93 ± 0.16	34.5	56.5	9.0	4.65 ± 0.22	0.24 ± 0.06	88.7	
S8	26	104	4.14 ± 0.19	46.2	40.6	13.2	4.43 ± 0.24	0.98 ± 0.07	82.1	
Clinical data of sampling sites										
S1	11	44	3.14 ± 0.13	83.7	14.0	2.3	3.51 ± 0.19	0.70 ± 0.10	67.4	1.02 ± 0.13
S2	13	52	4.16 ± 0.12	32.7	65.4	1.9	4.88 ± 0.19	0.84 ± 0.09	87.0	1.84 ± 0.13
S3	15	60	4.42 ± 0.27	45.8	28.8	25.4	5.29 ± 0.3	0.36 ± 0.07	63.0	1.17 ± 0.14
S4	14	56	4.07 ± 0.24	50.0	41.1	8.9	4.32 ± 0.24	0.54 ± 0.09	64.0	1.20 ± 0.14
S5	15	60	4.38 ± 0.22	41.7	41.7	16.7	4.38 ± 0.22	0.03 ± 0.02	80.0	1.62 ± 0.14
S6	14	56	4.16 ± 0.22	40.0	49.1	10.9	4.65 ± 0.3	0.58 ± 0.09	78.0	1.31 ± 0.12
S7	13	52	4.10 ± 0.14	33.3	58.8	7.8	4.49 ± 0.19	0.20 ± 0.06	88.0	1.47 ± 0.12
S8	12	48	3.96 ± 0.18	45.8	43.8	10.4	4.19 ± 0.23	0.96 ± 0.09	81.0	1.33 ± 0.13

*PD* probing depth, *CAL* clinical attachment level, *PI* plaque index, *BOP* bleeding on probing, *mSBI* modified sulcus bleeding index

### Levels of ODAM in GCF

**Fig. 1:**
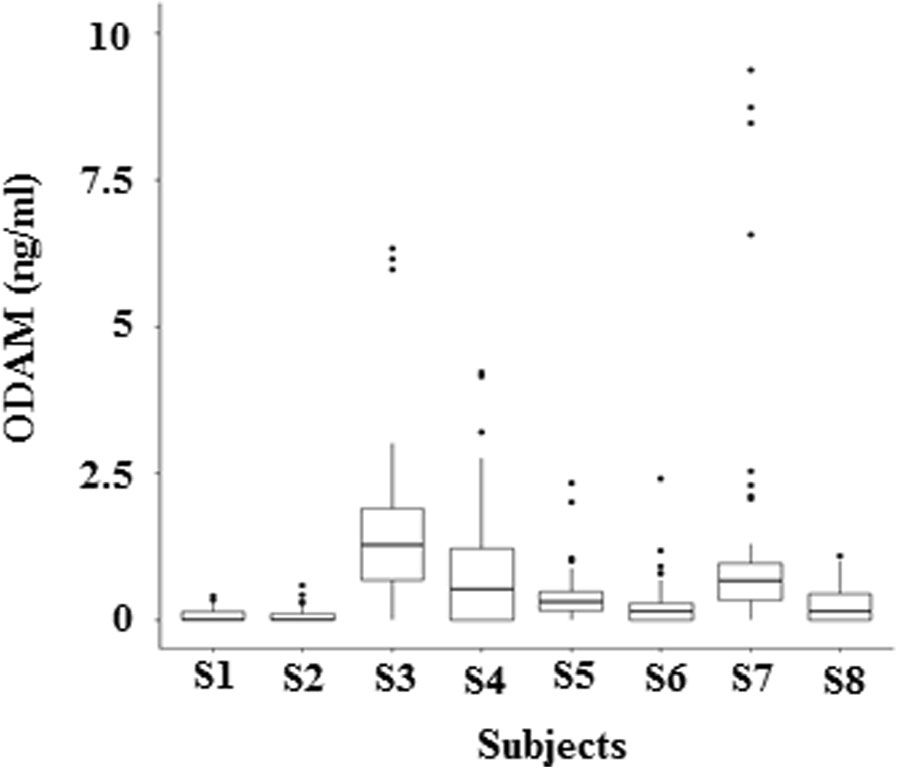
Box-plots showing the distribution of ODAM values in each individual patient (S1~S8). Eight periodontitis patients were sampled, and 40–56 GCF samples were analyzed from 11 to 15 teeth from each patient. Box plots show the medians, boxes represent the 25th and 75th percentiles, and black dots represent the 10th and 90th percentiles and outlier values

From the total of 428 GCF sites, four could not be processed properly in sampling or experimental steps, so a total 424 ODAM values in GCF were calculated from 8 subjects. In ELISA analysis, ODAM was not detected in 18.9% of the samples (80 among total 424 GCF samples) because the ODAM was below the limit of detection. In statistical analyses, these values were substituted with zero. Figure 1 illustrates the distributions of ODAM values in each individual patient. A broad range of inter-individual ODAM values was found, and the mean ODAM concentration for each periodontitis patient varied from 0.2 to 1.52 ng/ml of eluate (Fig. 1).

### Associations of the ODAM values in GCF with clinical parameters

**Fig. 2:**
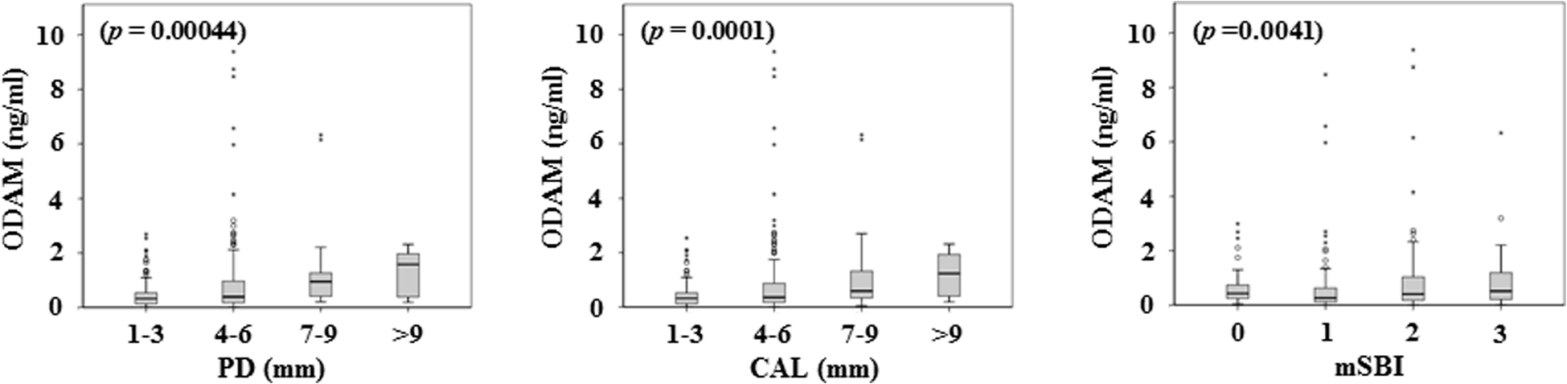
The ODAM value according to the severity of clinical parameters. *p*-values indicate the differences in ODAM values among groups classified by the severity of their clinical parameters (one-way ANOVA). Box plots show medians, boxes represent the 25th and 75th percentiles, and empty dots represent the 10th and 90th percentiles. Outlier values are shown as asterisks. PD; probing depth, CAL; clinical attachment level, mSBI; modified sulcus bleeding index

Correlations between ODAM values in GCF and clinical parameters were determined. ODAM values were significantly correlated with PD, CAL or mSBI (Table 2). The required sample size was calculated using a *p* value comparing PD with ODAM in GCF, the primary outcome of this study. A sample size of 193 achieves 80% power to detect a Spearman correlation of 0.214 using a two-sided hypothesis test with a significance level of 0.05. These result was based on 5000 Monte Carlo samples from the bivariate normal distribution under the alternative hypothesis [15]. When the dropout rate was considered to be 30%, the sample size was 276. Therefore, the analysis of total 424 sites was sufficient to prove the relationship between PD and the value of ODAM in GCF at site-specific level. The ODAM value according to the degree of clinical parameters was represented by a box plot, and the differences in ODAM values among groups classified by the severity of clinical parameters were analyzed by one-way ANOVA. The ODAM values showed a tendency to increase as the degree of clinical parameters increased. ODAM values were significantly different among groups divided by the severity of PD, CAL, or mSBI (PD; *p* = 0.00044, CAL; *p* = 0.0001, mSBI; *p* = 0.0041) (Fig. 2). Unadjusted and adjusted models were used to identify linear associations between PD, CAL or mSBI and ODAM in GCF. The unadjusted model showed significant linear associations between ODAM values and PD or CAL. Considering the GCF samples were collected from 44 to 60 sites from 11 to 15 teeth from each patient, the association between ODAM values in GCF and clinical parameters was analyzed using an adjusted linear mixed model adjusted within subject (as a random effect) and age, sex, plaque, and mSBI (as fixed effects). Significant associations between PD or CAL and ODAM values in GCF were also found through adjusted model (PD; β = 0.087, *p* = 0.026, CAL; β = 0.090, *p* = 0.005) (Table 3). Adjusted linear mixed model revealed that the ODAM value can be an indicator of PD or CAL. In sum, these findings suggest that raised ODAM levels in GCF can represent the degree of periodontal tissue destruction.

**Table 2:** Correlation of clinical parameters with ODAM in GCF

Clinical parameters	Correlation coefficient	*p*-value
PD	ρ = 0.214	< 0.0001
CAL	ρ = 0.232	< 0.0001
mSBI	ρ = 0.111	0.0216

*PD* probing depth, *CAL* clinical attachment level, *mSBI* modified sulcus bleeding index

**Table 3:** Association between ODAM values in GCF and clinical parameters

Variables	Unadjusted model			Adjusted model		
	β	SE(β)	*p*-value	β	SE(β)	*p*-value
PD^a^	0.091	0.039	0.019	0.087	0.039	0.026
CAL^a^	0.092	0.032	0.004	0.090	0.032	0.005
mSBI^b^	0.055	0.062	0.374	0.043	0.063	0.503

*PD* probing depth, *CAL* clinical attachment level, *mSBI* modified sulcus bleeding index, adjusted linear mixed model adjusted with subject (as a random effect) and age, sex, plaque, and mSBI (as a fixed effects)^a^ and age, sex, plaque(as a fixed effects)^b^

### ROC curve analysis

**Fig. 3:**
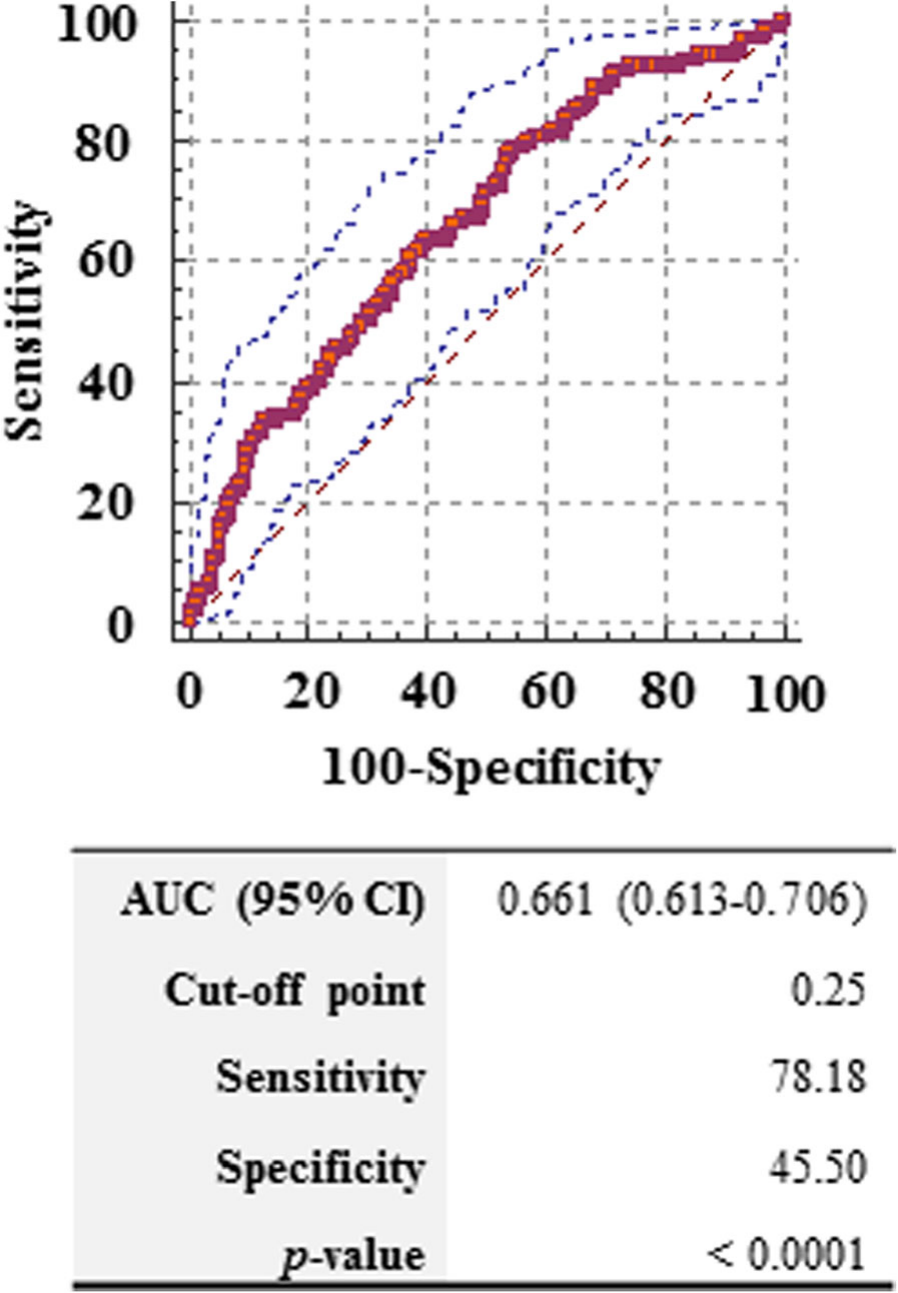
ROC analysis of ODAM values in GCF for the reflection of sites with PD ≥ 5 mm and positive BOP. ROC analysis for GCF ODAM to reflect the sites with PD ≥ 5 mm and positive BOP were constructed and areas under the receiver operating characteristic curve (AUC) were calculated to compare the predictive ability of the indices. Optimal cut-off values were determined using the Youden index to maximize the sum of sensitivity and specificity

The pocket with PD ≥ 5 mm is one of the most important risk indicators for periodontitis recurrence [16], therefore, the power of ODAM to reflect sites with PD ≥ 5 mm and positive BOP was evaluated by a ROC curve and the AUC. The AUC for GCF ODAM was 0.661, the 95% confidence interval was 0.613 to 0.706, and the *p* value was less than 0.0001 (Fig. 3). Optimal cut-off values were determined using the Youden index to maximize the sum of sensitivity and specificity. The cut-off value of 0.25 provided sensitivities of 78.18% and specificities of 45.50%.

## Discussion

This is the first study to assess the possibility of GCF ODAM as a site-specific biomarker for periodontitis. ODAM is involved in the adhesion of the JE to the tooth surface and is released into the gingival crevice when the adhesion is broken by progress into periodontitis [5]. It was analyzed whether there was a relationship between the value of ODAM in GCF and clinical diagnostic parameters at the same sites. As a result, ODAM appears to serve as a novel site-specific biomarker of periodontitis, as demonstrated by the statistically significant association between the value of ODAM in GCF and the parameters showing the degree of periodontal tissue destruction, PD or CAL. An adjusted linear mixed model showed that the ODAM value in GCF can be an indicator of PD or CAL. Our results suggest that ODAM in GCF plays a role as a site-specific biomarker for deep pockets, although additional studies including the change of ODAM values according to treatment are needed to determine the clinical significance of GCF ODAM. Since the loss of epithelial adhesion from the tooth surface is an early event in periodontal tissue destruction, it is worth verifying the potential of ODAM in GCF as a predictive biomarker for sites that may be vulnerable to periodontal bone loss. Based on the potential of ODAM as a predictive marker for the diagnosis of subjects (using saliva) and sites (using GCF) at risk for periodontitis, development of point-of-care diagnostic tools can help to overcome the limitations of current clinical diagnostics.

Periodontitis is developed at a site-specific level [17]. Therefore, GCF has been used as a tool for the diagnosis of periodontitis at a site-specific level because it reflects the site-specific severity of periodontitis. It contains a large number of proteins and peptides derived from underlying tissues. To date, nearly 100 different components in GCF have been reported as possible biomarkers for the progression of periodontitis [9, 12]. These include bacteria or bacterial products [18, 19], inflammatory mediators [20], host-derived enzymes and their inhibitors [21, 22] and soft and hard tissue destruction products [21, 23, 24]. Although there are many candidates, there has been no validation of factors involved in the attachment of the JE to the tooth surface. ODAM liberated from JE as a result of attachment loss can serve as an indicator of initial periodontal breakdown. Only extremely small volumes of fluid are available from a single site, so GCF requires highly sensitive techniques for quantitative analysis. In this study, ODAM was not detected in 18.9% of the samples. It is necessary to develop a highly sensitive and reliable detection tool that can detect ODAM at low concentrations through the development of a new antibody or aptamer.

The function of ODAM might be related to dentogingival attachment [5–7]. In this study, the value of ODAM in GCF was increased in deep periodontal pockets. This means that pathologic JE can also express ODAM after detachment from the tooth surface. However, in our previous study, ODAM was obviously expressed in the normal JE of healthy teeth but was absent in the pathologic pocket epithelium of diseased periodontium [5]. ODAM expression was reduced in the JE of experimental periodontitis by drugs, dextran sulfate sodium or periodontopathic bacteria (*Porphyromonas gingivalis*) compared with the sham group. Moreover, ODAM was not detected in the pocket epithelium of teeth extracted from periodontitis patients [5]. It is not readily explained that ODAM, which was not expressed or expressed at low levels in the JE of gingival biopsies from periodontitis patients, was detected at relatively high levels in the GCF from sites with deep pockets. Regarding these results, Wazen et al. noted how the level of ODAM in GCF would be maintained even though it is no longer produced by the JE [7]. Although the precise mechanism behind the expression of ODAM in periodontitis is unknown, one possibility may be that the detached JE continues to produce ODAM to maintain homeostasis for attachment to the tooth surface, and the resulting ODAM is immediately released into the pocket as soon as it is produced. Therefore, the ODAM may not be observed in histologic specimens of periodontitis models. Since the total area occupied by the pathologic pocket epithelium capable of producing ODAM is increased in periodontitis [4], the value of ODAM can be increase in deep periodontal pockets. It is also possible that the histological examination of the expression of ODAM according to the severity of periodontitis is not sufficiently verified. In our previous study, the examination of ODAM in JE from human gingival tissue was performed on only one specimen of gingiva around teeth extracted due to periodontitis [5]. Regarding ODAM expression in the JE of periodontitis, Nakayama et al. showed that ODAM gene expression was increased in inflamed gingiva from patients with chronic periodontitis using DNA microarray [25]. Recently, they also reported that the expression of ODAM was increased not only at the early stage but also at the following stages in the inflammatory JE on gingival biopsy from an experimental periodontitis model induced by *P. gingivalis*. They also showed that the localization of ODAM was spread into the gingival epithelium in inflamed gingiva using human gingival tissues [26]. To solve these discrepancies, histological examination of the expression of ODAM according to the severity of periodontitis should be performed.

The ROC analysis and AUC calculations were used to assess the ability of the ODAM to reflect sites with PD ≥ 5 mm and positive BOP. Pockets with a PD ≥ 5 mm have clinical significance; when compared with PD < 3 mm, PD ≥ 5 mm represented a risk factor for tooth loss [16]. Land & Tonetti divided the risk of periodontitis recurrence according to the number of pockets with a PD ≥ 5 mm [27]. In the analysis based on total subjects, the AUC for ODAM was 0.661, and it was interpreted that the ODAM value in GCF at the least has the potential to serve as a site-specific marker of deep pockets. Additional studies are needed to overcome the low sensitivity and AUC. Considering that the concentration of ODAM in the GCF varies greatly among subjects, it is expected that more accurate cut-off points having high sensitivity and specificity will be determined through the analysis of ODAM from more periodontitis patients.

There are two distinct approaches with respect to reporting GCF mediator content and concentration: the first is to sample GCF for a fixed time period and then report the results either as ρg per 30-s sample or by using the concentration as calculated from the assay (ρg/ml per 30-s sample), and the second is to convert the concentration calculated from the assay back into a concentration based on the original GCF volume according to the Periotron data [28–30]. The second approach allows one to know the actual concentration of the mediator in the GCF; however, it can have the potential for error associated with GCF volume determination and calculation [28]. A recent review of clinical and technical considerations in the analysis of GCF mentioned that recent authors tend to sample for a fixed period of time (usually 30 s) and report according to the first option described above [28]. In this study, GCF had to be taken from 40 to 56 sites from each patient, and as it requires a lot of time to measure GCF volume, we adopted the method of reporting by a fixed time period. However, to exclude the possibility that the ODAM value is just reflecting GCF volumes in deep pockets, the relationship between the concentration of ODAM and GCF volume was analyzed from an additional 30 GCF samples collected from 6 independent patients. There was no correlation between the concentration of ODAM and GCF volume (*p* = 0.750). The correlation was analyzed using a regression model and Stata/SE 11.1.

A limitation of this study is that GCF samples were collected from sites that have the potential to affect one another in the tooth. The design of the experiments was such that GCF samples were collected from 4 sites from each tooth, and as such, the levels of ODAM in the GCF recovered from mesio (or disto)-buccal sites on a specific tooth cannot be considered to be completely independent from the levels of ODAM in the GCF recovered from the mesio (or disto)-lingual sites on the same tooth. This limitation can lead to errors when analyzing the correlation between the ODAM values in GCF and the clinical parameters. Even so, ODAM in GCF was closely associated with clinical parameters and this can indicate the possibility of ODAM being used as a site-specific marker of deep pockets. Analysis using the GCF samples obtained from completely independent sites can eliminate the related error and provide a more precise correlation. This limitation should be corrected in future studies.

The sample size was calculated that total 424 sites was sufficient to prove the correlation between PD and the value of ODAM in GCF at site-specific level, however the study has the limitation that GCF samples are collected from only 8 subjects. Moreover, the mean ODAM concentration for each periodontitis patient varied. In order to overcome the limitation and apply statistical research methods suitable for data structures, the association between ODAM values and clinical parameters was analyzed using adjusted linear mixed models adjusted with subject effect. As a result, the adjusted model showed that the ODAM value in GCF can be an indicator of PD or CAL.

## Conclusion

In this cross-sectional study of periodontitis patients having simultaneously clinically healthy sites and diseased sites, levels of GCF ODAM were evaluated, and the association between the ODAM values and traditional clinical indices, including PD and CAL, were verified. It was enough to confirm the possibility of ODAM as a site-specific biomarker for periodontitis. Based on the pilot study, additional studies should be conducted on a large number of patients with various clinical severity to verify the clinical significance of GCF ODAM.

## Abbreviations

AUC: Area under the curve
BOP: Bleeding on probing
CAL: Clinical attachment level
ELISA: Enzyme-linked immunosorbent assay
GCF: Gingival crevicular fluid
JE: Junctional epithelium
mSBI: Modified sulcus bleeding index
ODAM: Odontogenic Ameloblast-Associated Protein
PD: Probing depth
PI: Plaque index
ROC: Receiver-operating characteristic
